# Biological hazards of micro- and nanoplastic with adsorbents and additives

**DOI:** 10.3389/fpubh.2024.1458727

**Published:** 2024-11-22

**Authors:** Ah Reum Hong, Jin Su Kim

**Affiliations:** ^1^Division of Applied RI, Korea Institute of Radiological and Medical Sciences (KIRAMS), Seoul, Republic of Korea; ^2^Radiological and Medico-Oncological Sciences, University of Science and Technology (UST), Seoul, Republic of Korea

**Keywords:** microplastics, nanoplastics, heavy metals, chemical additives, biological effects

## Abstract

With the increased worldwide production of plastics, interest in the biological hazards of microplastics (MP) and nanoplastics (NP), which are widely distributed as environmental pollutants, has also increased. This review aims to provide a comprehensive overview of the toxicological effects of MP and NP on *in vitro* and *in vivo* systems based on studies conducted over the past decade. We summarize key findings on how the type, size, and adsorbed substances of plastics, including chemical additives, impact organisms. Also, we address various exposure routes, such as ingestion, inhalation, and skin contact, and their biological effects on both aquatic and terrestrial organisms, as well as human health. Additionally, the review highlights the increased toxicity of MP and NP due to their smaller size and higher bioavailability, as well as the interactions between these particles and chemical additives. This review emphasizes the need for further research into the complex biological interactions and risks posed by the accumulation of MP and NP in the environment, while also proposing potential directions for future studies.

## Introduction

With accelerated industrialization globally, plastics, widely used in packaging, construction, and other industrial sectors have been mass-produced since the 1950s (1, 2). The production and consumption of lightweight, convenient, and useful plastics are increasing exponentially worldwide owing to their low manufacturing cost, safety, and hygiene (1, 3, 4). Following the COVID-19 pandemic in 2020, global plastic production reached 390.7 million tons in 2021 and is projected to be 34 billion metric tons by 2050 (5, 6).

MP are generated from various environmental factors and human activities. They are transported, dispersed, and deposited by wind flow, direction, and precipitation in the atmosphere. Through the atmosphere, which serves as a major pathway for MP transportation, all environmental compartments, including freshwater and terrestrial environments, can be impacted by MP pollution (7, 8). MP can be released into the air from plastic recycling processes in industries and waste disposal, synthetic fibers in carpets and clothing, as well as from friction activities like tire wear, which is also known to be a source of MP emissions (9, 10).

Plastic waste introduced into the environment is broken down into MP over time due to physical, chemical, and biological factors such as microbial degradation, ultraviolet (UV) exposure, and physical abrasion (11). Plastic fragments can be classified into categories based on their size, including megaplastics (>100 mm), macroplastics (>20 mm), mesoplastics (5–20 mm) microplastics (<5 mm), and nanoplastics (1–1,000 nm) (12–16). Both MP and NP are considered serious environmental problem due to their persistence and potential to be ingested by various organisms (15).

The most produced plastic polymers include polypropylene (PP, 19.3%), low density polyethylene (PE-LD, 14.4%), polyvinyl chloride (PVC, 12.9%), and high density polyethylene (PE-HD, 12.5%), polyethylene terephthalate (PET, 6.2%), polyurethane (PUR, 5.5%), and polystyrene (PS, 5.3%), in that order (5, 11). Additionally, environmentally friendly plastics are estimated to account for approximately 9.8% of global production (5). Consequently, plastic waste has become widespread in the environment and has accumulated in aquatic ecosystems worldwide, from Antarctica to the deep oceans (4, 17–19). By 2016, an estimated maximum of 23 million metric tons (Mt), approximately 11% of global plastic waste, had reached the aquatic ecosystems. If plastic waste continues to increase, the amount of plastic waste entering the world’s aquatic ecosystems is predicted to reach 90 Mt/year by 2030 (19).

The number of studies on the toxicity of MP/NP has been increasing (20, 21). The MP/NP generated after plastic waste decomposition are continuously dispersed accumulated in the environment, exerting toxic effects on aquatic and terrestrial wildlife and on humans (1, 21, 22).

MP originate from both primary plastics, which are manufactured in small sizes, and secondary plastics, which are created through the fragmentation of larger plastic waste (23). Several studies have indicated that MP negatively impacts the reproductive and feeding functions of crustaceans such as oysters (24) and mussels (25, 26). Furthermore, MP have been found in the feces of gentoo penguins in Antarctica (27), and research has reported the first occurrence of MP in demersal sharks in the UK (28). These findings suggest that MP can traverse the food chain, posing serious health risks to organisms (29).

MP degrade in the environment through physical, chemical, and biological processes, resulting in the formation of NP. Due to their smaller size, NP are more easily ingested by aquatic organisms, which can lead to bioaccumulation and serious health impacts on these organisms (30). These NP can also bind with heavy metals (31, 32) and chemicals (33, 34), exhibiting harmful effects such as reproductive toxicity (35, 36), intestinal toxicity (37, 38), and neurotoxicity (39, 40). Additionally, there are research studies that have confirmed that Antarctic krill, when consuming MP labeled with fluorescent substances, break them down into NP during the digestive process through the action of digestive enzymes (41). This study indicates that when most organisms ingest MP, they effectively consume NP simultaneously. This suggests that MP can serve as a resource for the formation of NP.

Also, researchers worldwide are increasingly focusing on the toxicity of mixtures formed by the adsorption of MP/NP with the additives used in their production and pollutants in the environment. However, current knowledge in this area is limited. Therefore, we emphasize the need for more research to reveal the interactions and biological hazards of chemicals associated with MP/NP accumulating in the environment. Our review of literature published over the past 10 years revealed that research on the toxicity of MP as well as NP, which are smaller and potentially more harmful than the MP, has rapidly increased (42, 43). In this review, we emphasized the need for more; the findings from this review can contribute to conducting systematic research on the biological hazards of not only MP/NP but also composite compounds.

## Classification of biohazards of microplastics

We conducted a search for articles published from 2012 to 2022 in PubMed Central (PMC). To search for papers related to all types of MP, we conducted searches using both the abbreviations and full names. Examples of keywords used in the search included PS, polymethyl methacrylate (PMMA), PA, PE, PVC, PP, PET, and polylactic acid (PLA). Research articles related to the biological effects of MP, excluding those from an environmental perspective, reviews, and other types of articles such as editorial materials, were selected from the retrieved hits. To find papers related to the biological effects of MP from 2012 to 2022, data queried using keywords such as the abbreviations and full names of MP were classified using Microsoft Excel 2019 (Microsoft Corporation, Santa Rosa, California, United States). Briefly, the list of papers was filtered using the filter function to analyze the data and derive related figures based on the type and size of the plastic and the presence or absence of additives. Supplementary Figure S1 presents a schematic of the literature search and process for extracting numerical data related to the biological impact of plastics. Supplementary Figure S2 shows the number of papers published by year. The number of papers related to the microplastics has dramatically increased over the last 12 years (2012–2024). Between 2012 and 2022, a total of 7,899 papers were published. In 2023 alone, 4,085 papers were published. In the first half of 2024, 2,490 papers were published, and a similar number is expected for the second half, indicating that even more papers will likely be released by the end of the year.

### Current status of research on the biological effects of microplastics

**Figure 1:**
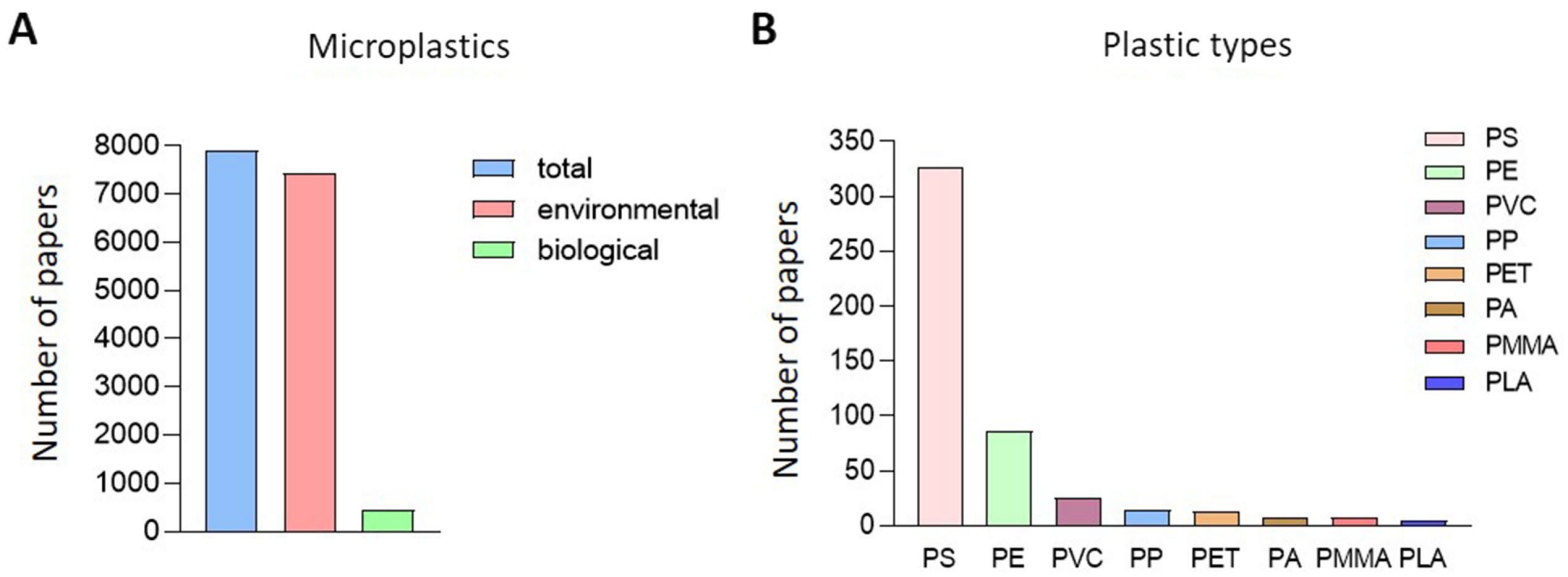
Research status of papers related to microplastics. **(A)** Overview of research on the biological effects of microplastics. **(B)** Classification of research by type of plastic.

**Figure 2:**
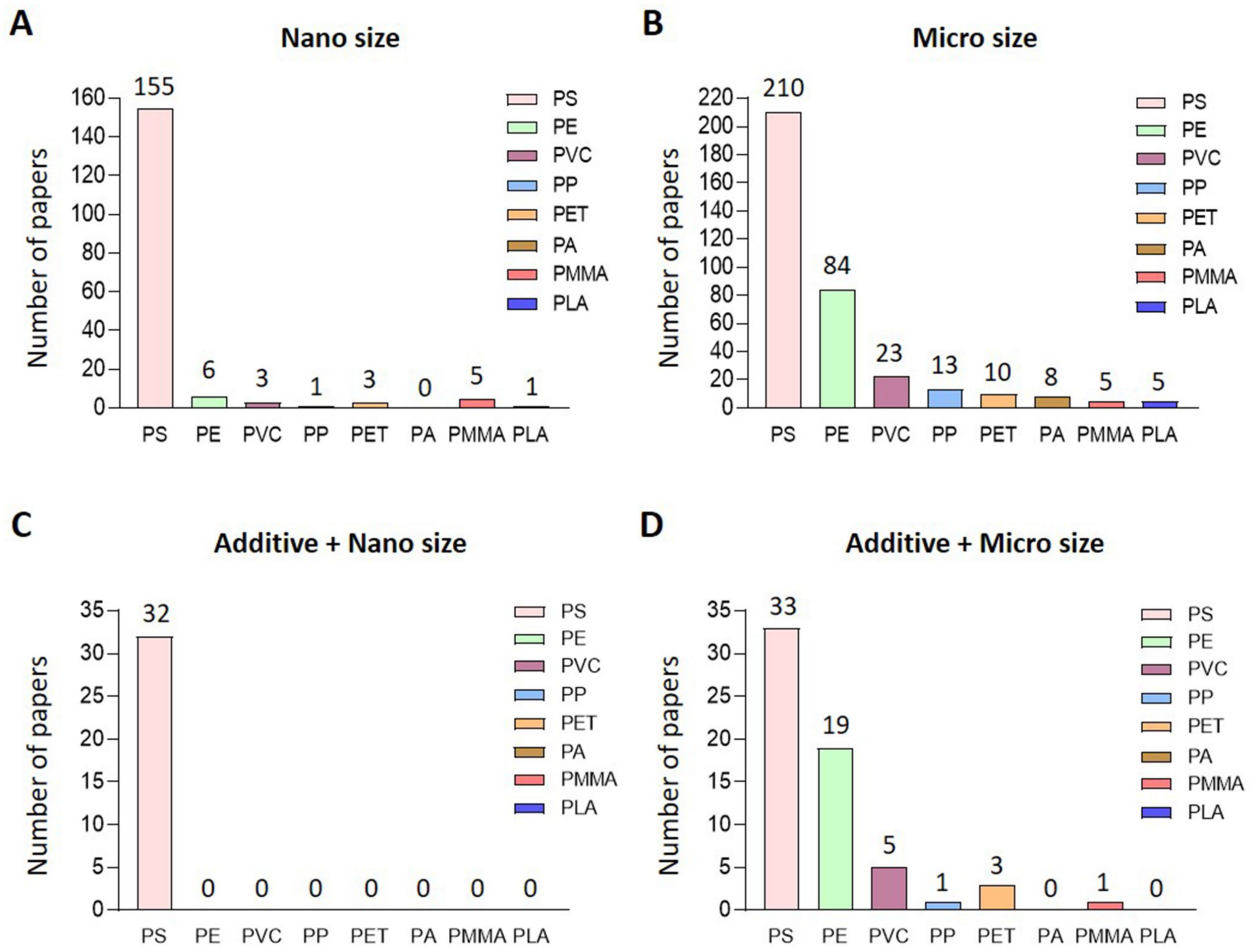
Current status of research on classification and additives according to size and type. Research classification of each type of plastic classified into **(A)** nano size and **(B)**. Micro size, **(C)** additive and nano sized, **(D)** additive and micro sized plastic.

Out of 7,899 papers searched for MP-related keywords in PubMed, 457 papers were related to biological impacts (Figure 1A). Among the studies focusing on the biological effects of MP, the most commonly studied plastic types were PS, PE, PVC, PP, and PET. Interestingly, research on PMMA, which is less frequently detected in natural ecosystems, has recently been conducted. On classifying 457 papers that evaluated biological hazards according to the type of MP, PS was the most common (326), followed by PE at 86, PVC at 25, and PS at 326. Fourteen articles on PP, 13 on PET, 8 on PA, 8 on PMMA, and 5 on PLA were published (Figure 1B). After classifying papers for NP of each type of plastic, there were 155 papers for PS, 6 for PE, 3 for PVC, 1 for PP, 3 for PET, 5 for PMMA, and 1 for PLA (Figure 2A). Regarding the MP, there were 210 cases for PS, 84 for PE, 23 for PVC, 13 for PP and 10 for PET. Also, there were published in 8 cases for PA, 5 for PMMA and 5 for PLA (Figure 2B). For papers evaluating the biological hazards of adding mixtures, such as heavy metals, according to the type and size of MP, 32 papers on PS were investigated for NP (Figure 2C). Among the NP, only PS has been used to evaluate biological hazards using mixtures, and the addition of mixtures to other types of NP has not yet been published. However, in studies evaluating the biological hazards of adding mixtures to MP, PS was the most common with 33, followed by PE with 19, PVC with 5, and PP was investigated in 1 article, PET in three articles, and PMMA in one article. No study has yet been conducted to evaluate the biological hazards of mixtures of microsized PA (MP-PA) and microsized PLA (MP-PLA) (Figure 2D). Based on an investigation of papers published on MP over the last decade, extensive research has been conducted to evaluate their biological hazards. Therefore, we summarized the type- and size-based characterization of MP *in vitro* and *in vivo* and the toxic effects of combined exposure to MP and additives, providing new evidence and insights into the potential biohazards of MP.

### Evaluation of the biological effects of micro/nanosized plastics on specific organs in biological samples

MP present in the environment can have harmful biological effects by penetrating not only fish and land mammals but also the human body by inhalation of air or consumption of food contaminated with MP. However, the unique characteristics and complexity of biological samples make the detection of MP in them more difficult than that in environmental samples. Therefore, we compiled an overview and analysis of methods used for detecting MP in recently published papers (Tables 1–4) with the aim of providing a reference for research exploring the distribution and characteristics of MP in biological samples from fish, terrestrial mammals, and humans. We conducted a recent literature search to compile information on the types, exposure durations, methods, and concentrations of MP detected in biological samples from fish, terrestrial mammals, and humans. We categorized the data based on the organs, including the skin, intestines, lungs, and brain, and summarized the associated biological effects.

**Table 1:** Summary of the biological effects of microplastics and nanoplastics on the skin.

Reference	Model	Polymer	Size	Route	Duration	Dose	Additive	Limitation	Actual result
(44)	Human-derived dermal fibroblasts (HDF cells)	PP	~20 μm, 25–200 μm	*In vitro*	48 h	10, 50, 100, 500, 1,000 μg/mL	x		Elevated ROS levels induced toxicity and larger particles demonstrated lower cellular toxicity
(45)	Human-derived dermal fibroblasts (HDF cells)	PS	PS (10, 40, 100 μm), PS-FITC (460 nm, 1 μm, 3 μm)	*In vitro*	24 h	0, 1, 10, 100, 500 or 1,000 μg/mL	x	Hemolytic impact of nano-sized PS was unknown	Small-sized PS particles induced hemolysis in red blood cells
(46)	Human-derived dermal fibroblasts (HDF cells)	PS	5–25 μm, 25–75 μm, 75–200 μm	*In vitro*	1 and 4 d	10, 100, 1,000 μg/mL	x		PS generated reactive oxygen species (ROS)
(47)	Human skin reconstructed 3D model	PP	86 μm	*In vitro* (3D-reconstructed epidermal tissue)	22 h	40 mg	x	The 3D reconstructed human skin model lacks pores	No cell toxicity
(48)	Skin keratinocytes in zebrafish embryos	PS	25 nm	*In vitro* (zebrafish embryos)	96 h	10, 25, 50 mg/L	x		PS induced mitochondrial damage and ionocyte apoptosis

**Table 2 tab2:** Summary of the biological effects of microplastics and nanoplastics on the intestine.

Reference	Model	Polymer	Size	Route	Duration	Dose	Additive	Limitation	Actual result
(49)	Adult zebrafish (*Danio rerio*)	PE	30 μm	Oral (in water)	7 d	1, 10, 100, 1,000 μg/mL	x		Altered intestinal microbiota activated the intestinal immune network
(50)	Mouse	PS	5 μm	Oral	42 d	100 μg/L	x		PS disrupts the homeostasis of colonic epithelium
(52)	Mouse	PS	40–60 μm, 60–100 μm	Oral	21 weeks	50, 500 mg/kg food	x	Significant PS accumulation was not observed in the intestine or liver	Imbalance in gut microbiota, tissue inflammation
(51)	Chickens (*Gallus gallus*)	PS	5 μm	Oral	6 weeks	1, 10, 100 mg/L	x	Lack of data that PS disrupt the intestinal barrier and penetrate into the gut	PS disrupts the intestinal vascular barrier, disturbs gut microbiota, and promotes the accumulation of lipids and carbohydrates
(38)	Human colonocytes (HT-29, Caco-2, CCD 841 CoN)	PS	100 nm	*In vitro*	24, 48, 72 h	PS-NP: 50, 100, 250, 500 μg/mL, F^−^: 1 mM	Fluoride	Intracellular internalization of PS in cells was conducted solely through TEM	Alterations in cellular microstructure

**Table 3 tab3:** Summary of the biological effects of microplastics and nanoplastics on the lung.

Reference	Model	Polymer	Size	Route	Duration	Dose	Additive	Limitation	Actual result
(39)	Mouse	PS	99 nm, 5 μm	Intranasal	5 weeks	10 μg/μL	x		Imbalance of microbiota
(53)	Human lung carcinoma, (A549)	PS	70, 200, 500 nm	*In vitro*	4, 10, 24 h	5, 15, 50, 150, 500, 1,500 mg/L	x		Internalized to cells
(54)	Human lung carcinoma, (A549l)	PE	7 μm (±2.0), 31 μm (±10.5)	*In vitro*	48 h	1, 10, 100, 500, 1,000 μg/mL	x		NO-mediated toxicity
(55)	Human alveolar type II epithelial cell line (A549)	PET	<20 nm	*In vitro*	24 h	1, 5, 10, 25, 50, 99, 197 μg/mL	x		Internalized to cells reactive oxygen species (ROS) detected
(56)	Mouse	PS	5 μm	Oral/intratracheal	7 d and 3 weeks	Oral administration: 100 mg/kg, intratracheal administration: 2, 7, 12 mg/kg	x	Assessment of the Wnt/β-catenin signaling pathway in mouse lung	Pulmonary epithelial damage

**Table 4:** Summary of the biological effects of microplastics and nanoplastics on the brain.

Reference	Model	Polymer	Size	Route	Duration	Dose	Additive	Limitation	Actual result
(57)	Zebrafish embryo	PS	1, 6, 10, 25 μm	*In vitro* (zebrafish embryos)	6 hpf (hours post-fertilization) ~ 120 hpf	500, 5,000, 50,000 particles/mL	x		Neurotoxicity (seizure effects)
(58)	Adult zebrafish (*Danio rerio*)	PE, PP	179 ± 77 nm	Oral (in water)	21 d	1 mg/L	x		Mitochondrial respiration deficiency
(59)	Common carp, (*Cyprinus carpio*)	PE	MaPs > 5 mm, 5 mm > MPs > 100 nm, NPs <100 nm	Oral (in water)	15 d	100 mg/L	x	Behavioral experiment data are needed to assess the neurotoxicity of PE	Neurotoxicity (decreased acetylcholinesterase (AChE), monoamine oxidase (MAO), and nitric oxide (NO))
(60)	Mouse	PS	1, 4, 10 μm	Oral	180 d	100, 1,000 μg/L	x	The underlying mechanisms of neurotoxicity and cognitive dysfunction induced by PS was unclear	The destruction of the blood-brain barrier, increased dendritic spine density, inflammation responses in the hippocampus, and impaired cognition and memory
(61)	Chicken	PS	5 μm	Oral	6 weeks	1, 10, 100 mg/L	x	Immunohistochemical staining methods to show clearly the presence of hemorrhage	Brain hemorrhage, loss of Purkinje cells

MaPs, macroplastics.

#### Skin

Table 1 summarizes the effects of microsized polypropylene (MP-PP) on skin cells. Human-derived dermal fibroblasts (HDF) were exposed to various concentrations of MP-PP for 48 h. MP-PP showed cytotoxicity in HDF, rodent macrophages (Raw 264.7), and human peripheral blood mononuclear cells (PBMC), linked to increased reactive oxygen species (ROS). Cytokine production (IL-6, TNF-α, histamine), associated with immune responses, also varied with particle size and concentration (44).

In another experiment, HDFs treated with various concentrations of microsized polystyrene (MP-PS) for 24 h showed particle penetration and accumulation within the cells. This induced the inflammatory cytokine IL-6, indicating potential local inflammation. Moreover, MP-PS infiltration into the cytoplasm triggered acute inflammatory responses in immune cells, increased ROS production, and released cytokines, leading to higher cell death in fibroblasts (45, 46). However, in an experiment using rodents and terrestrial mammals in which MP-PP was orally administered for 4 weeks, there was no toxicity or mutagenic potential. Additionally, 3D reconstructed human skin cell culture models showed no signs of skin irritation. This suggested that PP exposure does not have a negative effect on humans (47).

In a 96-h experiment with zebrafish embryos, exposure to nanosized polystyrene (NP-PS) reduced survival rates and damaged skin keratinocyte villi. Also, this exposure inhibited antioxidant responses, induced oxidative stress, caused mitochondrial damage, and led to ionocyte death, impairing ion uptake, pH regulation, and ammonia excretion (48). In conclusion, MP can induce cytotoxicity in skin cells, increase inflammatory cytokines, and trigger acute inflammatory responses in immune cells. Additionally, experiments using zebrafish embryos demonstrate that MP can reduce survival rates and damage skin keratinocytes.

#### Intestine

Table 2 summarizes the effects of plastics on the intestine. Human-derived colon cell lines (HT-29, Caco-2, and CCD 841CoN) were treated with nanosized polystyrene (NP-PS) at various times and concentrations. The results showed that PS was absorbed by colon cancer cells in a time- and concentration-dependent manner, leading to cytotoxicity. Specifically, HT-29 cells internalized PS, resulting in ultrastructural changes and cell death. Co-exposure to PS and F-further increased HT-29 cell death (38). The biological effects of exposing zebrafish to microsized PE (MP-PE) in water tanks were investigated. MP-PE reduced the range of intestinal goblet cells and altered the abundance of dominant microorganisms in the intestines. This exposure also activated intestinal immune network pathways responsible for mucosal immunoglobulin production (49). In experiments using terrestrial mammals, such as rodents and chickens, MP-PS was provided as drinking water and food, and its biological effects on the intestines were evaluated (50–52). In a mouse colitis model, PS disrupted colonic epithelium, induced liver inflammation, and exacerbated colitis, suggesting long-term exposure to PS poses significant health risks despite no significant accumulation in intestinal tissues (50). In chickens exposed to MP-PS in drinking water, PS damaged the intestinal vascular barrier, disrupted intestinal flora, caused intestinal necrosis, and induced inflammatory cell death (pyroptosis) due to microbial infections. Additionally, PS triggered hepatic immune responses, leading to lipid metabolism disorders and cell death in the liver (51). In mice, PS exposure primarily caused gut microbiota dysbiosis, tissue inflammation, and plasma lipid metabolism disorders, without significant PS accumulation in the intestines or liver. Gut microbiota changes were closely related to PS concentration and size, while intestinal damage and abnormal lipid metabolism were not significantly linked to PS exposure (52). In conclusion, plastics induce cytotoxicity in a size-dependent manner, with smaller sizes leading to internalization and subsequent cell death. Furthermore, MP has been shown to reduce the range of goblet cells and alter gut microbiota composition in zebrafish. In mammals, they cause damage to the colonic epithelium, liver inflammation, and disruption of gut microbial communities, which may result in gut microbiota dysbiosis and tissue inflammation.

#### Lung

Table 3 shows results for the biological effects of plastics on lungs. Research using human lung carcinoma cells (A549) investigated the biological effects of different-sized PS, PE, and PET particles (53–55). PS could be internalized by cells through phagocytosis, and could the findings facilitate the understanding of health risks caused by such accumulation (53). At 1,000 μg/mL, PE slightly reduced A549 cell viability and induced high levels of nitric oxide (NO) and nitrite. This suggests PE exposure may increase susceptibility to NO-mediated toxicity and immune modulation (54). PET was internalized by A549 cells, reducing cell viability at high concentrations and inducing oxidative stress. Increased PET concentrations correlated with decreased mitochondrial membrane potential and higher levels of reactive oxygen species (ROS), leading to an increase in late-stage apoptotic cells (55). In experiments with terrestrial mammals, intranasal administration of NP-PS and MP-PS to mice induced nasal microbiota imbalance, with MP-PS showing a stronger effect on lung microorganisms. Suggesting microbial changes could serve as biomarkers for PS-induced airway imbalance (39). Inhalation exposure to intratracheal PS via spray induced dose-dependent pulmonary fibrosis in mice, increasing α-SMA, vimentin, and Col1a expression. This exposure also caused intensive lung oxidative stress, suggesting PS inhalation may lead to pulmonary fibrosis through oxidative stress and Wnt/β-catenin signaling activation (56). In conclusion, plastic particles can be internalized by cells, increasing health risks, reducing the viability of lung cells, and inducing oxidative stress while affecting immune modulation. Additionally, in mammals, nanosized plastics have been shown to disrupt lung microbiota balance, and inhalation exposure may lead to pulmonary fibrosis, potentially linked to oxidative stress.

#### Brain

Table 4 summarizes the search results for the biological effects of plastics detected in the brain. Zebrafish embryos exposed to MP-PS exhibited seizures, increased seizure-like brainwave signals, and altered seizure-related gene expression. PS disrupts cholinergic, dopaminergic, and GABAergic neurotransmitter systems, impacting brain development in zebrafish embryos (57). In experiments with adult zebrafish, exposure to NP-PE and NP-PP in aquariums increased brain catalase activity but inhibited lactate dehydrogenase at high doses. Brain respiratory chain complexes II and IV significantly decreased, indicating impaired mitochondrial function. In the liver, mitochondrial respiration was also impaired, correlating with decreased mitochondrial membrane potential due to respiratory chain complex inhibition (58). In an experiment with common carp (*Cyprinus carpio*), exposure to NP-PE and MP-PE in aquariums significantly reduced acetylcholinesterase (AChE), monoamine oxidase (MAO), and nitric oxide (NO) levels in the brain. Smaller PE particle sizes correlated with more pronounced reductions in these markers. Additionally, damage such as necrosis, fibrosis, capillary changes, tissue disintegration, edema, and degenerative connective tissue was observed in cerebellar neurons, ganglion cells, and the retina, indicating potential neurotoxic effects of PE exposure (59). Furthermore, an experiment in terrestrial mammals investigated the effects of consuming MP-PS in drinking water. It found that PS disrupted the blood-brain barrier, increased brain dendritic spine density, and induced hippocampal inflammation. Mice exposed to PS exhibited impaired cognition and memory, with concentration-dependent effects on learning abilities, irrespective of PS particle size (60). In experiments with chickens exposed to MP-PS in drinking water, significant effects on the brain were observed, including hemorrhage, microthrombi formation, and loss of Purkinje cells. Plastic-induced brain hemorrhage triggered inflammation, disrupted mitochondrial function, and activated signaling pathways like ASC-NLRP3-GSDMD and AMPK (61). In conclusion, plastics impact neurotransmitter systems in zebrafish embryos, while they increase brain catalase activity and inhibit lactate dehydrogenase in adult zebrafish. In common carp, exposure to plastics leads to decreased levels of acetylcholinesterase (AChE), monoamine oxidase (MAO), and nitric oxide (NO). Furthermore, in mammals, the blood-brain barrier is disrupted, resulting in impaired cognition and memory, with significant observations in chickens, including brain hemorrhage and loss of Purkinje cells.

## Assessments of biological effects of combined exposure to micro/nanoplastics and additives

Recently, the number of studies evaluating the biological hazards of mixtures containing MP and heavy metals or additives, rather than focusing solely on the biological toxicity of individual MP substances, is increasing. In this section, findings from studies that have assessed the biological effects of mixtures involving MP/NP of varying sizes and types combined with heavy metals or additives are summarized.

### A summary of the combined exposure of nanoplastics with heavy metals or additives

#### Nanoplastics with heavy metals

Chen et al. (62) demonstrated that co-exposure of cadmium (Cd) and NP-PS in grass carp (*Ctenopharyngodon idellus*) decreases antioxidant enzyme activity and causes organ damage. Feng et al. (31) showed that co-exposure to MP-PS and lead (Pb) in female mice aggravated ovarian toxicity and increased Pb bioaccumulation. Estrela et al. (63) demonstrated that zinc oxide nanoparticles (ZnO) in combination with NP-PS influenced the behavior of *Ctenopharyngodon idella* (*C. idella*) in mirror tests, inducing inactivity and showing signs of DNA damage and increased oxidative stress. In mice, Estrela et al. (40) evaluated the toxicity of ZnO and NP-PS through intraperitoneal administration, revealing increased levels of nitric oxide (NO), reactive oxygen species, decreased acetylcholinesterase (AChE) activity, and brain accumulation of nanomaterials, indicating their potential neurotoxicity. Yu et al. (32) used single-cell sequencing to reveal heterogeneous effects of NP and Pb on zebrafish intestinal cells. Simultaneous exposure to NP-PS and Pb altered immune recognition, induced cell death, and caused oxidative stress, lipid metabolism disturbance, and similar intestinal toxicity.

#### Nanoplastics with chemicals

Steckiewicz et al. (38) demonstrated that fluoride alone was not cytotoxic but enhanced the cytotoxicity of NP-PS in colonocytes, causing ultrastructural changes through cellular internalization. Yu et al. (37) demonstrated that co-exposure to MP/NP and oxytetracycline in zebrafish led to altered intestinal histopathology, microbiome changes, and increased antibiotic-resistance genes. Li et al. (35) found that NP-PS enhanced the adverse effects of di-(2-ethylhexyl) phthalate (DEHP) on the male reproductive system in mice, causing gene and pathway alterations. Also, Liao et al. (64) showed that DEHP exacerbates the toxicity of NP-PS through histological damage and intestinal microbiota dysbiosis in freshwater fish. Wang et al. (65) demonstrated the Simultaneous exposure to NP-PS and BDE-47 in zebrafish exacerbated developmental deformities, decreased survival rates, and caused tissue damage. Santos et al. (66) demonstrated combined exposure to NP and phenmedipham (PHE) in zebrafish embryos exhibited greater toxicity than single exposures. Martínez-Álvarez et al. (67) showed that combined exposure to NP-PS and benzo(a)pyrene (B(a)P) in brine shrimp larvae and zebrafish embryos increased toxicity. Qin et al. (68) showed that chlorine disinfection increased NP-PS toxicity in human cells by inducing mitochondria-dependent apoptosis. Liu et al. (69) showed that NP and avobenzone (AVO) exposure affected neural and retinal development in zebrafish. Liu et al. (70) demonstrated NP-PS and butyl methoxydibenzoylmethane (BMDBM) affected zebrafish brain development and inhibited motor activity. Wu et al. (34) showed that parental co-exposure to NP-PS and microcystin-LR (MCLR) aggravated hatching inhibition in zebrafish offspring, affecting enzyme activity, disrupting the cholinergic system, and impairing muscle development. Similarly, Zuo et al. (71) demonstrated that combined exposure to NP-PS and MCLR altered the expression of HPT axis-related genes and GH/IGF axis genes in F1 zebrafish larvae, exacerbating growth inhibition and increasing MCLR transfer to offspring. Wang et al. (33) demonstrated the NP-PS and bisphenol A (BPA) in human cells showed increased adsorption and cytotoxicity. Singh et al. (72) demonstrated that NP-PS and polycyclic aromatic hydrocarbons (PAHs) altered nanoparticle stability and toxicity, leading to DNA damage in zebrafish. He et al. (36) demonstrated co-exposure to NP and triphenyl phosphate (TPhP) in zebrafish led to significant reproductive impairment. Yan et al. (73) demonstrated NP and tetracycline (TC) in gastric cancer cells reduced cell viability and induced oxidative stress. Zhang et al. (74) showed NP-PS combined with roxithromycin (ROX) in freshwater fish red tilapia (*Oreochromis niloticus*) increased bioaccumulation and disrupted metabolism. Chen et al. (75) showed that NP and 17α-ethynylestradiol (EE2) exposure in zebrafish suppressed locomotor activity and altered swimming behavior. Bhagat et al. (76) demonstrated that co-exposure to NP and metal oxide nanoparticles (nMOx) like aluminum oxide and cerium oxide induced oxidative stress in zebrafish embryos. Zhao et al. (77) showed that NP-PS and synthetic phenolic antioxidants like butylated hydroxyanisole (BHA) in zebrafish disrupted thyroid function and metabolism.

#### Nanoplastics with others

Alaraby et al. (78) demonstrated antagonistic interactions between silver compounds and NP-PS in *Drosophila*, where nanosilver, known for inducing oxidative stress, significantly decreased oxidative stress and DNA damage when combined with NP-PS, thereby reducing genotoxicity. In contrast, Ilić et al. (79) showed a synergistic interaction between silver nanoparticles (AgNP) and NP-PS in human intestinal cells, with combined exposure leading to increased cell death, expression of inflammatory cytokines (IL-6, IL-8, and TNF-α), oxidative stress, and mitochondrial dysfunction. Guo et al. (80) showed that NP-PS significantly altered the gut microbial community in zebrafish, with commercial PS having stronger toxic effects, which were mitigated by co-treatment with enrofloxacin (ENR). Brandts et al. (81) demonstrated the immunomodulatory effects of NP and humic acids on European seabass. The study assessed whether NP-PS act as stressors in juvenile European seabass, affecting immune response, and whether humic acid mitigates these effects. Shi et al. (82) found that NP-PS and phthalate esters together reduced cell viability in human lung epithelial A549 cells, emphasizing their combined toxic effects and risks of co-exposure to NP and organic pollutants in humans. Hou et al. (83) showed significant NP accumulation in human intestinal organoids, investigating their absorption, toxicity in human intestinal cells, and proposing inhibiting intracellular uptake as a potential therapy to reduce NP toxicity in humans.

In conclusion, combined exposure to NP and heavy metals or additives has been shown to increase cytotoxicity, resulting in reproductive and intestinal toxicity, as well as organ damage. This exposure leads to heightened DNA damage and oxidative stress, which in turn contributes to increased inflammation. Additionally, alterations in gut microbial communities have been observed.

## A summary of the combined exposure of microsized plastics with heavy metals or additives

### Microsized polystyrene

#### Microsized polystyrene with heavy metals

Wang et al. (84) reported distinct adverse outcomes on erythrocytes’ lipid profiles following single and combined exposure to Cd and MP. Co-treatment of MP-PS and CdCl2 showed a clear antagonistic relationship, indicating impaired membrane function of red blood cells (RBCs). Chen et al. (85) demonstrated that co-exposure to MP-PS and Cd in early-stage zebrafish reduced body weight and intensified growth abnormalities, oxidative stress, and cell death-related gene expression compared to individual exposures. These findings suggest that MP may worsen Cd’s adverse effects during early zebrafish development. Zhang et al. (86) studied the combined toxicity of MP and Cd in zebrafish embryos. They exposed the embryos to varying concentrations of MP along with environmentally relevant levels of Cd, which adversely affected their survival and heart rate (HR). Yan et al. (87) showed that the individual and combined toxicogenetic effects of MP and heavy metals (Cd, Pb, and Zn) disrupted gut microbiota and gonadal development in marine medaka. This affected gut function and specific bacterial species in male fish. Lu et al. (88) found that MP increase Cd levels in zebrafish organs, including the liver, viscera, and gills. This combined exposure to MP and Cd resulted in increased toxicity, leading to oxidative damage and inflammation. The study emphasizes the chronic risks of MP and Cd exposure in zebrafish. Zuo et al. (89) demonstrated the individual and combined effects of MP and Cd on juvenile grass carp (*Ctenopharyngodon idellus*). They found that intestinal Cd levels were elevated in grass carp exposed to both Cd and MP-PS. Histological analysis showed significant intestinal damage following acute exposure, accompanied by changes in proinflammatory cytokine expression. Yang et al. (90) compared the combined toxicity of MP-PS and different Cd concentrations in zebrafish. They found that MP-PS increased Cd toxicity at low concentrations (LCd) but reduced toxicity at high concentrations (HCd), indicating a concentration-dependent interaction between MP-PS and Cd in zebrafish. Zhang et al. (91) found that combined exposure of goldfish to MP and Cu induced oxidative stress, inflammation, apoptosis, and impaired autophagy in the pancreas and intestines. MP enhanced Cu accumulation in the liver, pancreas, and intestines, worsening oxidative stress. This combined exposure also leads to inflammation, excessive cell death, and impaired autophagy in the liver and pancreas, further emphasizing the risks associated with MP-mediated heavy metal adsorption. Zheng et al. (92) found that particles, rather than Zn^2+^ released from ZnO nanoparticles, exacerbated MP toxicity in early-stage exposure in zebrafish and their offspring. ZnO particles attached to MP surfaces facilitated ZnO transport into larvae, intensifying effects on growth inhibition, oxidative stress, apoptosis, and GH/IGF axes.

#### Microsized polystyrene with chemicals

Yu et al. (37) showed that combined exposure to MP/NP and oxytetracycline in zebrafish affected intestinal histopathology and microbiome. Co-exposure increased antibiotic resistance gene abundance in the intestine. Cheng et al. (93) studied the combined effects of MP-PS and BPA on human embryonic stem cell-derived liver organoids, highlighting metabolism-related health risks even at low doses equivalent to human internal exposure levels. Wang et al. (33) studied the combined effects of BPA and NP-PS, MP-PS on particle uptake and toxicity in human Caco-2 cells. They examined how BPA adsorbs onto different sizes of PS particles using colon cancer Caco-2 cells and assessed resulting cell toxicity, confirming increased toxicity with BPA adsorption during MP exposure. Sun et al. (94) discovered that simultaneous ingestion of MP-PS and epoxiconazole increases health risks in mice due to synergistic bioaccumulation. Intestinal damage caused by EPO allows significant PS penetration, impacting gut microbiota and exacerbating oxidative stress and metabolic disorders. Lu et al. (95) investigated the combined toxicity of MP-PS and sulfamethoxazole (SMZ) on zebrafish embryos. Despite observing an antagonistic effect between PS and SMZ toxicity, which slightly reduced their combined impact, co-exposure still exhibited significant toxicity. Jiang et al. (96) investigated the effects of MP and tributyltin (TBT), alone and combined, on bile acid and gut microbiota interactions in mice. They observed that MP, either alone or with TBT, induced liver inflammation, altered gut microbiota composition, and disrupted fecal bile acid profiles. However, combined exposure to MP and TBT mitigated the toxic effects observed with individual exposures. Domenech et al. (97) showed the interaction of silver nanoparticles and silver nitrate with PS as metal carriers and their effects on human intestinal Caco-2 cells. In this study, we confirmed that a composite of silver compounds and PS was internalized by Caco-2 cells, exhibiting harmful cellular effects, such as genetic toxicity and oxidative DNA damage. Xu et al. (98) investigated the toxic effects of MP and phenanthrene in zebrafish. Combined exposure led to higher accumulation in zebrafish and increased expression of immune and oxidative stress genes due to oxidative stress. MP also demonstrated a synergistic effect by altering gut microbiota, thereby enhancing the toxicity of phenanthrene. He et al. (36) demonstrated enhanced toxicity of triphenyl phosphate (TPhP) in zebrafish when combined with MP and NP. MP-PS was used to study TPhP toxicity, revealing that MP further inhibited sperm and oocyte formation and significantly impaired zebrafish reproductive performance compared to TPhP alone. Li et al. (99) showed that hydrogen sulfide (NaHS) mitigates MP-PS-induced hepatotoxic effects by upregulating the Keap1-Nrf2 pathway. NaHS significantly reduced inflammation, cell death, and oxidative stress in the liver caused by MP-PS, promoting Nrf2 accumulation and alleviating its hepatotoxic effects. Yang et al. (100) discovered that MP-PS reduced 6:2 chlorinated polyfluorinated ether sulfonate (F-53B) bioaccumulation in larval zebrafish while promoting its adsorption, thereby lowering its bioavailability. This combined exposure also induced inflammatory stress in the zebrafish larvae. Wang et al. (101) showed that MP and DEHP together induced pancreatic cell apoptosis in mice through oxidative stress and activation of the GRP78/CHOP/Bcl-2 pathway. This study showed increased ROS levels, inhibited antioxidant enzyme activity, and altered expression of key pathway proteins, ultimately leading to cell death. Hanslik et al. (102) studied biomarker responses in zebrafish (*Danio rerio*) exposed long-term to MP-bound chlorpyrifos (CPF) and benzo(k)fluoranthene (BkF). They found that CPF, an organophosphate insecticide, adsorbed onto MP-PS during exposure to adult zebrafish, while BkF, a polycyclic aromatic hydrocarbon (PAH), adsorbed onto microsized polymethyl methacrylate (MP-PMMA). Importantly, these MP-bound substances did not induce adverse effects in aquatic ecosystems. Luo et al. (103) showed that exposure to both MP-PS and imidacloprid (IMI) in adult zebrafish led to enhanced liver toxicity by affecting genes involved in glycolipid metabolism and inflammation. This highlights the synergistic hepatotoxic effects of MP and IMI in zebrafish.

#### Microsized polystyrene with others

Qiao et al. (104) explored the combined effects of MP-PS and natural organic matter (NOM) on Cu accumulation and toxicity in zebrafish. They found that smaller MPs absorbed more Cu, and NOM facilitated Cu adsorption onto MPs. This combination increased Cu accumulation in the liver and gut in a size-dependent manner, suggesting heightened Cu toxicity in these organs. Deng et al. (105) demonstrated that MP worsen the toxicity of organophosphorus flame retardants (OPFRs) in mice. Co-exposure increased lactate dehydrogenase levels and decreased AChE activity, alongside significant metabolic changes in amino acid pathways and energy metabolism compared to controls. Zhang et al. (74) demonstrated that MP-PS interact with ROX to enhance its bioaccumulation and distribution in freshwater red tilapia. Co-exposure to PS and ROX potentially affects ROX metabolism in the liver of red tilapia. Zhao et al. (77) showed that microplastics worsened the developmental toxicity of synthetic phenolic antioxidants in zebrafish by disrupting thyroid function and metabolism, leading to increased BHA accumulation, lower hatching rates, more deformities, and reduced bone calcification. Yan et al. (73) investigated the toxicity of TC in combination with PS spheres in gastric cancer cells. They confirmed that PS had a concentration-dependent adsorption capacity for TC using two different sizes of PS. Moreover, the PS-TC mixture reduced the viability of gastric cancer cells (AGS) by inducing oxidative stress, ultimately leading to cell death.

In conclusion, the combined exposure of MP with heavy metals or additives has been shown to increase cytotoxicity, negatively impact erythrocytes, and induce developmental abnormalities. Furthermore, this combined exposure may enhance toxicity through accumulation in organs, leading to intestinal damage and alterations in gut microbial communities.

### Microsized polyethylene

#### Microsized polyethylene with heavy metals

Tarasco et al. (106) studied the effects of pristine and contaminated MP-PE on zebrafish development, finding impaired reproductive capacity with BaP and MP-PE co-exposure. They noted increased skeletal deformities and bone disorders during development, alongside intestinal inflammation indicated by histological analysis. Banaee et al. (107) investigated the effects of Cd and MP particles on common carp (*Cyprinus carpio*), focusing on biochemical and immunological parameters. They found that combined exposure to Cd and MP reduced lysozyme and alternative complement (ACH50) activity, as well as total immunoglobulin and complements C3 and C4 levels compared to controls. These changes indicated heightened toxicity on immunological parameters. Miranda et al. (108) investigated the neurotoxic, behavioral, and lethal effects of Cd, MP, and their mixtures on juvenile *pomatoschistus microps* under lab conditions. They found that while mortality rates did not significantly differ between groups exposed to Cd alone versus the MP-Cd mixture, MP did influence the sublethal neurotoxic effects of Cd. Luís et al. (109) demonstrated that MP influence the acute toxicity of chromium (Cr) (VI) in early juvenile common gobies (*Pomatoschistus microps*). They found a significant decrease in predatory performance and inhibition of AChE activity when juveniles were co-exposed to Cr (VI) and MP-PE. Additionally, confriming that co-exposure led to increased lipid peroxidation (LPO).

#### Microsized polyethylene with chemicals

Menéndez-Pedriza et al. (110) investigated the lipidomic impacts of MP-PE and PCBs on human hepatoma cells. They found that while MP-PE alone was non-toxic, its combination with PCBs led to concentration-dependent changes in lipid composition and membrane permeability, indicating potential adverse effects from this interaction. Huang et al. (111) investigated the combined impact of MP and tetrabromobisphenol A (TBBPA) on the human gut using *in vitro* simulations with human colon cancer cells and microbial communities. They found that this combined exposure disrupted gut homeostasis and metabolic pathways in gut microbiota, leading to significant adverse effects. Yu et al. (112) found that cosmetic-derived plastic microbeads enhance TBBPA adsorption and increase oxidative stress in zebrafish. The integrated biomarker response (IBR) index revealed significant detrimental effects from combined PE and TBBPA exposure. Zhang et al. (113) studied the combined effects of PE and 9-nitroanthracene (9-NAnt) on zebrafish, finding that this co-exposure induced neurotoxicity, disrupted energy metabolism, and altered gut microbiota composition. Deng et al. (114) demonstrated that phthalate-contaminated MP increased PAE accumulation in the liver and intestine of male mice, leading to enhanced reproductive toxicity. Combined exposure to PAEs and MP adversely affected sperm physiology and formation. Deng et al. (52) found that MP adsorb and transport PAEs to the mouse intestine, where they accumulate. Combined exposure to MP and PAEs increased intestinal permeability, altered gut microbiota, and exacerbated inflammation and metabolic disorders more than individual exposures. Sheng et al. (115) demonstrated that different types of MP affect triclosan (TCS) adsorption, accumulation, and toxicity in zebrafish. PE increased TCS accumulation in the liver and intestines by adsorbing TCS. Combined exposure to TCS and PE led to increased lipid toxicity due to TCS accumulation. Deng et al. (105) showed that MP worsen the toxicity of organophosphorus flame retardants (OPFRs) in mice. Mice exposed to MP-PE along with OPFRs like TCEP and TDCPP experienced more pronounced changes in biochemical markers and metabolites compared to exposure to these substances individually, indicating increased toxicity from the combined exposure. Tong et al. (116) demonstrated that MP-PE cooperate with *Helicobacter pylori* to promote gastric injury and inflammation in mice. In this study, exposure to a combination of *Helicobacter pylori* and MP-PE resulted in increased infiltration of inflammatory cells into gastric or intestinal tissues, along with an elevation in inflammatory factors.

#### Microsized polyethylene with others

Khan et al. (117) found that MP-PE beads did not significantly change silver (Ag) absorption and localization in zebrafish. However, MP-PE increased Ag accumulation in the intestines, suggesting alterations in the bioavailability and absorption of metal contaminants. Boyle et al. (118) demonstrated that PVC plastic fragments release bioavailable Pb additives in zebrafish. They compared the impact of PVC exposure with PE-HD and PET exposure, noting that PE did not significantly alter biomarker expression. Schirinzi et al. (119) studied the cytotoxic effects of nanomaterials and MP on human cerebral and epithelial cells. They assessed individual cell toxicity for PE, metal nanoparticles (nMOx), and carbon nanomaterials, observing heightened oxidative stress in both cell lines. This suggests that combined exposure to PE and these additives induces cellular toxicity. Batel et al. (120) found that PE transferred BaP to *Artemia nauplii* and zebrafish in a food web experiment. PE moved through zebrafish intestines without causing significant damage but was absorbed by epithelial cells. It also facilitated the release of persistent organic pollutants (POPs) in the intestines, transferring them to the intestinal epithelium and liver. Araújo et al. (121) examined the combined effects of emerging pollutants and MP-PE on zebrafish, focusing on genotoxicity and redox balance. They found that both MP-PE alone and in combination with new pollutants caused DNA damage and nuclear abnormalities in erythrocytes. This indicates that the combined exposure did not increase toxicity beyond that of MP-PE alone, highlighting complex interactions among substances in aquatic environments. Batel et al. (122) investigated the long-term ingestion effects of differently sized MP on zebrafish. Their study focused on BaP combined with PE, demonstrating that BaP-PE particles accumulated in the zebrafish intestine but particles were transported along the intestine and excreted without inducing adverse effects.

In conclusion, the combined exposure of MP with heavy metals and additives has been shown to enhance cellular toxicity and induce developmental disorders, neurotoxicity, and dysbiosis in gut microbiota. Additionally, as bioaccumulation within the body increases, it may lead to organ damage and alterations in the bioavailability of these substances.

### Microsized polyvinyl chloride

#### Microsized polyvinyl chloride with heavy metals

Chen et al. (123) used the SBRC (Soluble Bioavailability Research Consortium) digestion model to study the bioaccessibility of heavy metals (As, Cr, Cd, Pb) associated with MP and PVC. They found that Pb (II) exhibited higher bioaccessibility compared to As (V), Cr (VI), and Cd (II), highlighting potential health risks related to the interactions between heavy metals and MP. Boyle et al. (118) demonstrated that PVC plastic fragments release bioavailable Pb additives in zebrafish. Their study assessed the effects of PVC and Pb additives on zebrafish biomarker expression, confirming that MP-PVC serves as an environmental reservoir for Pb, impacting biomarkers. Hoseini et al. (124) demonstrated severe hepatic stress and inflammation in common carp (*Cyprinus carpio*) exposed to copper (Cu) and MP-PVC. The combined exposure induced significant liver damage and inflammation, as evidenced by hepatic transcriptomic and histopathological responses.

#### Microsized polyvinyl chloride with chemicals

Wang et al. (125) demonstrated that single exposure to MP-PVC and DEHP delayed hatching and caused mortality in juvenile zebrafish. Single exposure affected cardiac development, while combined exposure showed an antagonistic effect. Sheng et al. (115) studied the impact of different MP types on triclosan (TCS) adsorption, accumulation, and toxicity in zebrafish. They observed PVC’s ability to adsorb both forms of TCS, altering its tissue distribution and increasing TCS accumulation in the liver and intestines. This highlights potential harmful effects of PVC-TCS mixtures on zebrafish.

In conclusion, combined exposure to MP with heavy metals and additives has been shown to induce significant hepatic stress and inflammation, leading to liver damage. Furthermore, this exposure may also result in developmental disorders and has the potential to adsorb and accumulate specific substances in the liver and intestines, which could pose harmful effects.

### Microsized polypropylene

Sheng et al. (115) studied the impact of different MP types, including PP, on triclosan (TCS) adsorption, accumulation, and toxicity in zebrafish. They found that MP-PP had the highest TCS adsorption capacity, leading to increased accumulation in the liver and intestines. Co-exposure to MP-PP and TCS also heightened oxidative stress, lipid peroxidation in the liver, and neurotoxic effects in the brain.

### Microsized polyethylene terephthalate

Boyle et al. (118) found that PVC plastic fragments release bioavailable Pb additives in zebrafish. They studied both PVC and microsized polyethylene terephthalate (MP-PET) regarding Pb release in zebrafish. The study concluded that PET did not alter biomarker expression in zebrafish larvae, indicating it had no effect on Pb release. Liu et al. (126) found that MP-PET reduced the bioaccumulation of SMZ in various tissues of mice but worsened its effects on gut microbiota and antibiotic resistance genes. While SMZ levels in the liver, lungs, spleen, heart, and kidneys were lower with MP-PET, the interaction exacerbated impact of SMZ on gut microbiota and antibiotic resistance gene profiles. Cheng et al. (127) studied the effects of MP fibers and granules on zebrafish embryos, both alone and in combination with Cd. They found that PET granules (p-PET) increased blood flow velocity and heart rate and inhibited embryo hatching, while PET fibers (f-PET) reduced Cd accumulation in the chorion by dissolving in the culture medium. Overall, both p-PET and f-PET decreased Cd toxicity, with fibers showing a stronger detoxification effect.

### Microsized polymethyl methacrylate

Hanslik et al. (102) studied biomarker responses in zebrafish exposed long-term to MP-associated chlorpyrifos (CPF) and benzo(k)fluoranthene (BkF). They found that BkF adsorbed onto MP-PMMA in zebrafish, and combined exposure reduced BkF bioavailability compared to exposure to BkF alone, suggesting no adverse effects from PMMA-bound BkF in zebrafish.

## Discussion

In this review, we set a 10-year period from 2012 to 2022 and conducted a comprehensive search for relevant studies in PubMed. We identified any biological hazards depending on the size and type of plastic and classified and organized the results of mixed exposure with chemicals and heavy metals included during plastic preparation as well as numerous chemicals pre-exposed in the environment.

Hazard tests conducted on various organs, including the skin, intestine, lungs, and brain, to assess the biological effects of exposure to MP/NP revealed that in the skin they inhibit antioxidant responses, induce oxidative stress, and lead to cell death, potentially damaging skin function (45–48). In the intestines, they alter the microflora, cause tissue inflammation, destruct the vascular barrier, and cause metabolic disorders (38, 50–52). In the lungs, they cause an increase in oxidative stress and an imbalance in nasal microorganisms, potentially causing lung fibrosis (39, 53, 54, 56). Finally, in the brain they impact the regulatory disorders associated with seizures. They could cross the blood-brain barrier, induce inflammatory responses in the hippocampus, and trigger inflammatory cell infiltration into the brain as a result of brain hemorrhage, potentially leading to intracerebral inflammation (57, 60, 61). These findings suggest that the toxic effects induced by MP/NP could be significant, potentially reaching humans at the top of the food chain (128).

Majority of the global plastic is produced for use as packaging material in food, cosmetics, and pharmaceuticals (2, 129). Plastics are composed of numerous compounds including various chemicals. When manufacturing plastics for specific purposes, various chemical additives such as lubricants, plasticizers, antioxidants, heat stabilizers, and pigments are used during production and formulation (20, 130, 131). Plastics manufactured by incorporating numerous chemicals, when exposed to various environments, decompose into MP and cause biological hazards because of their ability to adsorb contaminants from the surrounding environment (93, 94, 110, 114, 122, 132).

DEHP, a commonly used plasticizer enhances the toxic effects on the male reproductive system when simultaneously exposed with plastics (35), causing histological damage microbial imbalance in the intestine (52, 64). Similarly, BPA, an endocrine-disrupting substance, has been used as an additive to render plastics transparent. Concurrent exposure to BPA and plastics increases metabolism-related hazards in human embryonic stem cell-derived liver organoids and can cause diseases (93). Simultaneous exposure to plastics and PBDE, which are used as flame retardants in plastics and fabrics, induces morphological developmental disorders, damages muscle and cartilage tissues, and exacerbates toxic effects on the thyroid (65). Additionally, BHA, an antioxidant widely used in plastics, food, and cosmetics, accumulates in plastics, disrupts thyroid function and metabolism, and worsens developmental toxicity (77).

Heavy metals such as Pb, Cd, Al, and ZnO are also used during plastic manufacturing and exist in a relatively stable form within plastics (133). However, studies have also demonstrated that MP/NP break down into small particles (134), and adsorb heavy metals present in the surrounding environment. These composite compounds have been shown to induce various side effects and diseases (76, 92, 108, 124). Co-exposure to Pb and plastics increases Pb accumulation in the ovaries of female mice, exacerbating ovarian toxicity (31). In aquatic organisms such as zebrafish (*Danio rerio*), they induced toxicity and immune recognition disorders in intestinal epithelial cells (32), and disrupted intestinal microbial homeostasis and reproductive development (87). Cd co-exposure with plastics causes damage to the gills, kidneys, liver, and muscles of aquatic organisms (62) and has negative effects on growth, survival, and heart rate (85). Moreover, when Al is co-exposed with plastics, they inhibit efflux pumps and induce oxidative stress in zebrafish embryos (76). When co-exposed with plastics, another heavy metal, ZnO, can cause DNA damage in zebrafish (72), leading to growth inhibition and cell death (92). In mice, the accumulation of nanomaterials in the brain because of co-exposure results in cognitive impairments (40). A variety of models, including aquatic organisms, higher terrestrial organisms, and human-derived cells, have been utilized in such research, and the experimental results varied depending on the size and type of plastics and additives (44, 52, 93, 118).

In this review, we searched PMC for papers related to MP over the past 10 years. Through this, we were able to visualize comprehensive information on the current status of MP research. According to our data, many publications over the past 10 years have confirmed the growing interest of researchers in MP/NP. MP/NP, which exist after plastic waste enters the environment and decomposes into fragments of various sizes, are already exposed to living organisms through oral ingestion, inhalation, or skin contact. Many studies have been conducted to date on the hazards caused by these various exposure routes. In addition, research has shown that decomposed plastic fragments can combine with various adsorbents, such as various surrounding chemicals or heavy metals, and that these composite compounds can cause more toxic reactions than the previously known harmful effects of MP/NP. However, research on the toxicity mechanisms of MP/NP is limited, and studies on the toxicity of composite compounds formed by various adsorbents are either biased towards specific sizes or types or lack sufficient evidence for established results.

Adsorbents and additives play a crucial role in shaping the fate and toxicity of MP and NP. This review critically assesses how different types of adsorbents and additives influence the bioavailability, persistence, and transport of plastics in various environments. Additionally, we explore the potential synergistic or antagonistic effects that may arise from the combination of plastics with different adsorbents and additives.

Building on the existing body of knowledge, this review proposes a new understanding that synthesizes the complex interactions between MP and NP, adsorbents, additives, and biological systems. By acknowledging the multifaceted nature of these interactions, we aim to move beyond a simplistic view of plastic pollution and biological hazards. This nuanced perspective allows for a more accurate assessment of risks and the formulation of targeted mitigation strategies. The added value of this review lies in its synthesis of disparate research findings, offering a comprehensive and up-to-date overview of the biological hazards associated with micro- and nano-plastics in the presence of adsorbents and additives.

By establishing a new position that considers the interplay of multiple factors, this review provides a roadmap for future research, guiding scientists, policymakers, and stakeholders toward more effective and sustainable solutions for mitigating the impacts of plastic pollution. This review contributes to the evolving discourse on the biological hazards of MP and NP by providing a nuanced understanding of the role of adsorbents and additives. By recognizing the complexities inherent in these interactions, we pave the way for targeted research efforts and informed decision-making to address the challenges posed by plastic pollution.

This review emphasized the need for further research to understand and establish the biological hazards of MP/NP and their interactions with plastic additives and different chemical substances in the environment. This review will provide researchers around the world with an understanding of the interactions of MP/NP with additives and suggests new research directions.

## Conclusion

Accordingly, it is expected that this paper will contribute to active research on the toxicity mechanisms of MP/NP, or the toxic effects of composite compounds that have not been revealed to date.
