# Supplementary figures and images for: Biological hazards of micro- and nanoplastic with adsorbents and additives
Source: Front Public Health. 2024 Nov 22;12:1458727. doi: 10.3389/fpubh.2024.1458727 (PMC11621061; doi:10.3389/fpubh.2024.1458727)

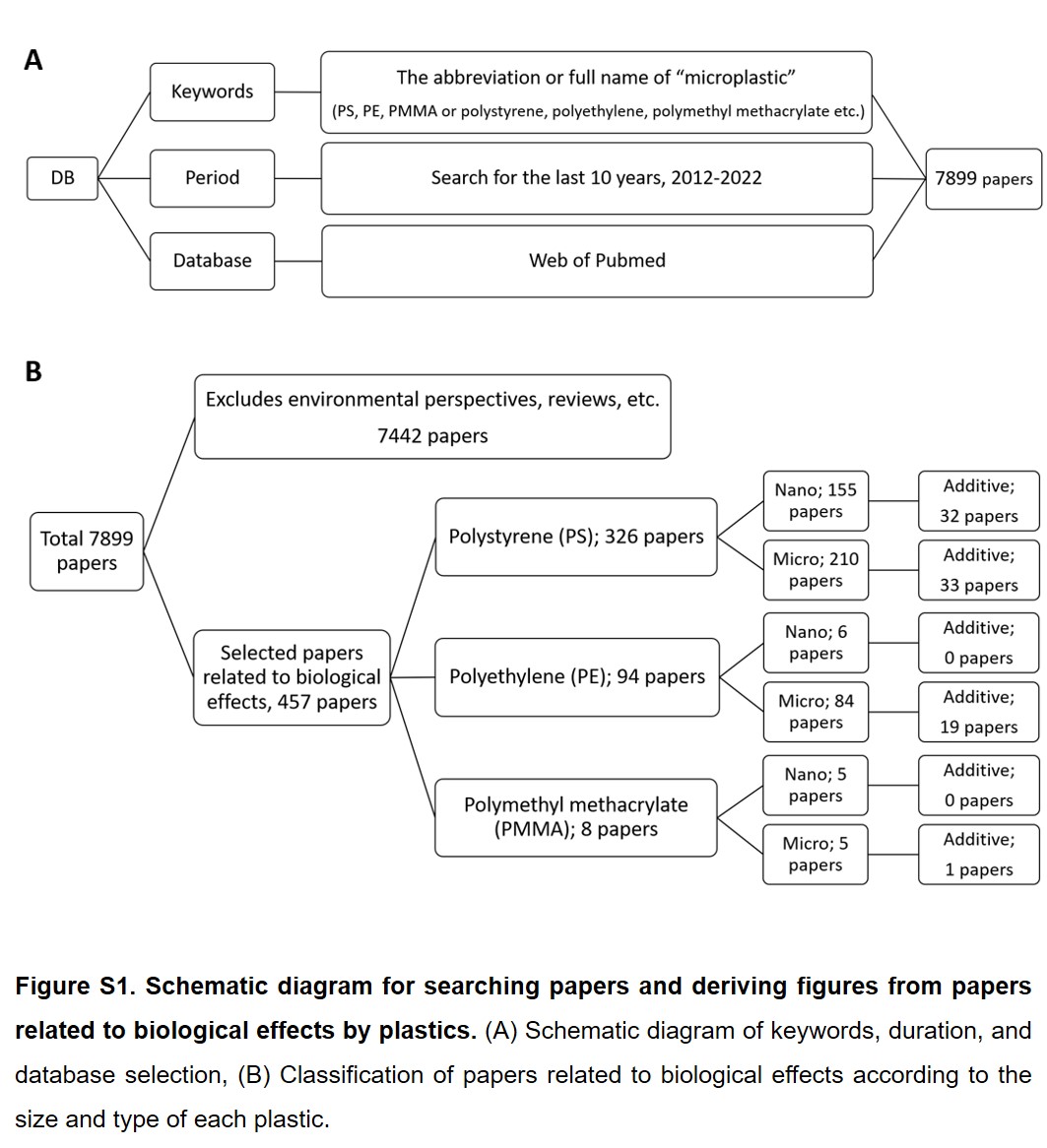

Supplement: Supplementary file 1 [file Image_1.JPEG]

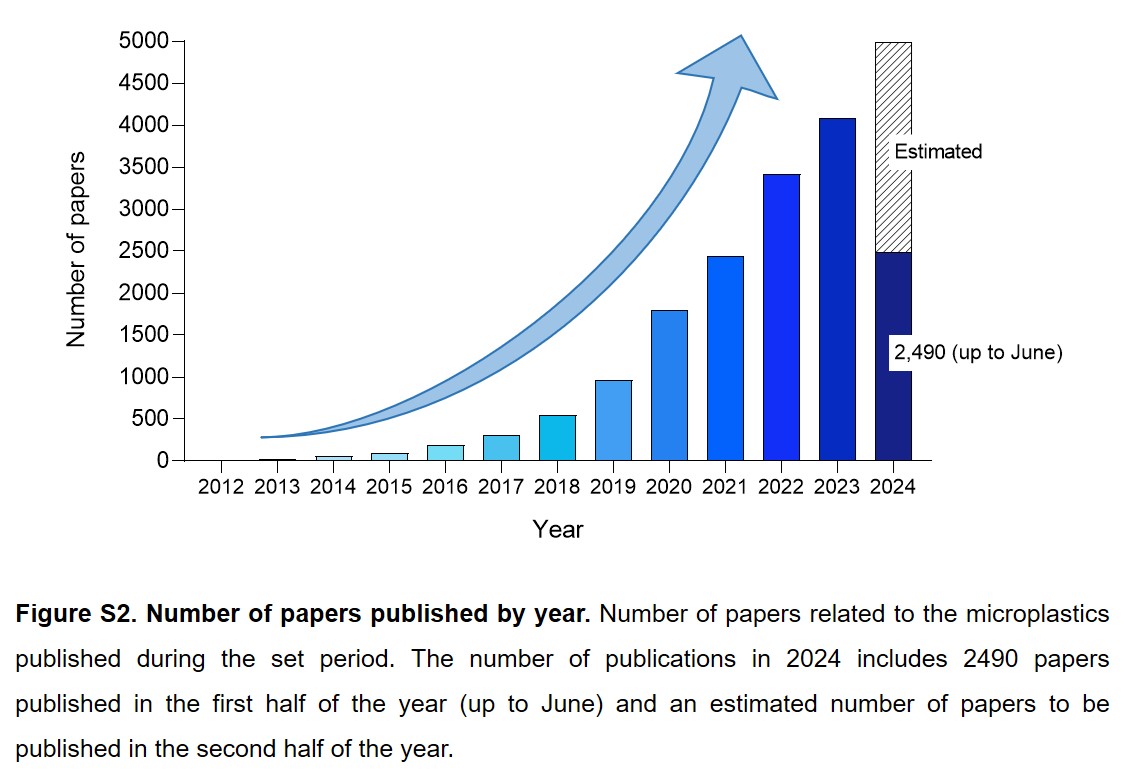

Supplement: Supplementary file 2 [file Image_2.JPEG]
